# Expression of *AOX1* Predicts Prognosis of Clear Cell Renal Cell Carcinoma

**DOI:** 10.3389/fgene.2021.683173

**Published:** 2021-07-05

**Authors:** Luyang Xiong, Yuchen Feng, Wei Hu, Jiahong Tan, Shusheng Li, Hongjie Wang

**Affiliations:** ^1^Department of Critical Care Medicine, Tongji Hospital, Tongji Medical College, Huazhong University of Science and Technology, Wuhan, China; ^2^Division of Pulmonary and Critical Care Medicine, Department of Internal Medicine, Tongji Hospital, Tongji Medical College, Huazhong University of Science and Technology, Wuhan, China; ^3^Department of Urology, Renmin Hospital of Wuhan University, Wuhan, China; ^4^Department of Obstetrics and Gynecology, Tongji Hospital, Tongji Medical College, Huazhong University of Science and Technology, Wuhan, China; ^5^Division of Cardiology, Department of Internal Medicine, Tongji Hospital, Tongji Medical College, Huazhong University of Science and Technology, Wuhan, China

**Keywords:** clear cell renal cell carcinoma, biomarker, *AOX1*, data mining, robust rank aggregation

## Abstract

Clear cell renal cell carcinoma (ccRCC) is the most prevalent kidney cancer worldwide, and appropriate cancer biomarkers facilitate early diagnosis, treatment, and prognosis prediction in cancer management. However, an accurate biomarker for ccRCC is lacking. This study identified 356 differentially expressed genes in ccRCC tissues compared with normal kidney tissues by integrative analysis of eight ccRCC datasets. Enrichment analysis of the differentially expressed genes unveiled improved adaptation to hypoxia and metabolic reprogramming of the tumor cells. Aldehyde oxidase 1 (*AOX1*) gene was identified as a biomarker for ccRCC among all the differentially expressed genes. ccRCC tissues expressed significantly lower *AOX1* than normal kidney tissues, which was further validated by immunohistochemistry at the protein level and The Cancer Genome Atlas (TCGA) data mining at the mRNA level. Higher *AOX1* expression predicted better overall survival in ccRCC patients. Furthermore, *AOX1* DNA copy number deletion and hypermethylation were negatively correlated with *AOX1* expression, which might be the potential mechanism for its dysregulation in ccRCC. Finally, we illustrated that the effect of *AOX1* as a tumor suppressor gene is not restricted to ccRCC but universally exists in many other cancer types. Hence, *AOX1* may act as a potential prognostic biomarker and therapeutic target for ccRCC.

## Introduction

According to 2020 GLOBOCAN data, an estimated 431,288 people worldwide were diagnosed with kidney cancer in 2020, accounting for 2.2% (431,288 cases) of all new cancer cases and 1.8% (179,368 deaths) of all new cancer deaths (Sung et al., 2021). Clear cell renal cell carcinoma (ccRCC) is the most common type of kidney cancer, making up 75% of all kidney cancer cases (Ricketts and Linehan, 2018). The 5-year overall survival decreases dramatically from 86% for stage I ccRCC to 18% for stage IV ccRCC in the United States (Feng et al., 2019). Typical ccRCC patients have symptoms such as hematuria, flank pain, and abdominal masses. However, most ccRCC patients are asymptomatic and diagnosed when undergoing imaging examinations, such as computed tomography scan or ultrasound (Padala et al., 2020). The lack of available and effective diagnostic methods precludes recognition and treatment of ccRCC patients at an early stage, leading to poor prognosis and low overall survival. Cancer biomarkers are biochemical molecules produced by tumor cells or normal cells of the organ or tissue in response to tumorigenesis. They facilitate accurate screening, early diagnosis, and prognostic prediction of cancer patients, which improve the outcome of ccRCC patients. However, a reliable biomarker for ccRCC has not yet been identified.

With the rapid development of next-generation sequencing, numerous sequencing data of ccRCC patients have been generated in the past 20 years. However, the integrative analysis of different sequencing datasets is difficult due to heterogeneity in sequencing platform, study design, and downstream data processing, which necessitates a robust method for joint analysis of multiple datasets. This study implemented the robust rank aggregation method *via* the RobustRankAggreg package in R, which consolidates differential analysis results from various datasets in an unbiased manner (Kolde et al., 2012). Eight ccRCC RNA sequencing (RNA-seq) datasets were incorporated and analyzed using the RobustRankAggreg package in R, and 356 differentially expressed genes between ccRCC tissues and normal kidney tissues were identified, including the aldehyde oxidase 1 (*AOX1*) gene.

According to the UniProt database, AOX1 is an oxidase with a broad spectrum of substrates that include multifarious aromatic azaheterocycles and aldehydes. In addition, it participates in the bioactivation of prodrug, regulation of oxygen species homeostasis, production of nitric oxide, and adipogenesis. Loss of AOX1 in advanced bladder cancer promoted cancer progression, and AOX1-related metabolites predicted advanced bladder cancer (Vantaku et al., 2020). Similarly, AOX1 is also involved in the development of colorectal cancer by transcriptional activation of CD133 *via* the phosphoinositide 3-kinase (PI3K)/Akt pathway (Zhang W. et al., 2020). Moreover, *AOX1* gene expression level correlates with recurrence of prostate cancer (Li et al., 2018). However, there is a paucity of data on the role of AOX1 in ccRCC.

This study identified shared differentially expressed genes across eight ccRCC RNA-seq datasets, analyzed enriched pathways, and established a protein–protein interaction (PPI) network for the differentially expressed genes. Then, we focused on AOX1’s effect on ccRCC. We assessed the differential expression of the *AOX1* gene in ccRCC tissues and normal kidney tissues and further validated its differential expression with immunohistochemistry and data mining in The Cancer Genome Atlas (TCGA) database. Furthermore, we proposed a potential mechanism for its dysregulation in ccRCC. The associations between *AOX1* gene expression and main clinical factors were evaluated. In addition, we profiled *AOX1* expression patterns in various normal human tissue and many other different cancer types and explored the AOX1-related disease spectrum.

## Materials and Methods

### Data Acquisition and Integrated Differential Expression Analysis

Gene Expression Omnibus (GEO), NCBI’s public functional genomics data repository, was thoroughly queried for datasets related to ccRCC. The inclusion criteria are as follows: (1) Datasets contain human ccRCC tissue samples. (2) Raw sequencing data are available for each sample. (3) Datasets contain normal kidney tissues as control. Gene expression data in each dataset were downloaded, imported, and analyzed using R (version 4.0.3). The limma package was used to identify differentially expressed genes in each study (Ritchie et al., 2015). And genes with absolute log_2_FC > 1 and adjusted *p*-value < 0.05 were defined as differentially expressed genes compared to the control group in a dataset. The robust rank aggregation method, which is parameter-free and robust to outliers, noise, and errors, was implemented to screen shared differentially expressed genes across all datasets (Kolde et al., 2012).

### Gene Ontology and Kyoto Encyclopedia of Genes and Genomes Pathway Analysis

To determine the biological processes, molecular functions, cellular components, and pathways relevant to the differentially expressed genes, Gene Ontology (GO) term enrichment and Kyoto Encyclopedia of Genes and Genomes (KEGG) pathway analyses were implemented using clusterProfiler package, which supports both hypergeometric test and gene set enrichment analysis for many ontologies and pathways (Yu et al., 2012).

### Protein–Protein Interaction Network Establishment

STRING^1^ is a database of known and predicted PPI that incorporates both physical and functional associations between proteins. We used STRING to build a PPI network from common differentially expressed genes across all datasets and Cytoscape^2^ software to visualize the network. The MCODE plugin (Bader and Hogue, 2003) in Cytoscape was used to find the functional modules in the PPI network.

### Survival Analysis of Differentially Expressed Genes

The Kaplan–Meier plotter is an online tool capable of assessing the survival effect of 54k genes on a wide spectrum of cancer types, which integrates cancer sources from GEO, European Genome-phenome Archive, and TCGA Program (Nagy et al., 2018). We estimated the survival effect on ccRCC patients of all selected hub genes using the Kaplan–Meier plotter, and log-rank test was applied to compare the survival distributions of different groups. Hazard ratio (HR) with 95% confidence interval was calculated when there was a difference.

### Gene Expression Profiling Interactive Analysis and Human Protein Atlas Profiling

Gene Expression Profiling Interactive Analysis (GEPIA) is a Web server for cancer and normal gene expression profiling based on TCGA and Genotype-Tissue Expression (GTEx) data (Tang et al., 2017). We used GEPIA to profile target gene expression in different cancer types. The Human Protein Atlas (HPA^3^) database provides a comprehensive map of all the human proteins in cells, tissues, and organs. We explored the expression of target gene at the protein level in different human tissues with the help of HPA database.

### Evaluation of Target–Disease Association

The Open Targets Platform is a robust data integration for access to and visualization of potential drug targets associated with numerous diseases (Carvalho-Silva et al., 2019). Associations between target gene and diseases were evaluated using the Open Target Platform.

### Data Mining in the Cancer Genome Atlas Kidney Renal Clear Cell Carcinoma Program

The level 3 data in The Cancer Genome Atlas Kidney Renal Clear Cell Carcinoma (TCGA-KIRC) dataset were acquired by UCSC Xena browser (Goldman et al., 2020). Only ccRCC patients with gene expression data were included in this study. ccRCC patients with a history of neoadjuvant treatment and without clear pathologic stage and histologic grade were excluded from this study. Extracted patient data include gender, pathologic stage, histologic grade, lymph node invasion, metastasis, overall survival, overall survival time, and genomic data of each TCGA-KIRC case. The genomic data include RNA-seq data of total gene expression, exon expression, methylation status, and DNA copy number alterations (CNAs) of a target gene. Cox regression analysis was used to evaluate the effect of different factors on ccRCC patients’ overall survival.

### Immunohistochemistry

Formalin-fixed, paraffin-embedded ccRCC tissues were subjected to immunohistochemical analysis to estimate the expression of AOX1. Briefly, 10-μm cross section was deparaffinized and rehydrated in a series of xylene and graded ethanol solutions. Heat-induced antigen retrieval was performed in Tris-EDTA buffer (pH 9.0, G1203; Servicebio, China) in a 99°C water bath for 15 min. Intrinsic peroxidase activity was deactivated by incubation with hydrogen peroxide for 30 min at room temperature. After blocking with 5% goat serum in phosphate-buffered saline (PBS) for 1 h at room temperature, ccRCC tissue sections were incubated with AOX1 antibody (1:100 dilution in PBS, 19495-1-AP; Proteintech, China) overnight at 4°C. Isotype control of primary antibody was used as a negative control to differentiate non-specific background signal. Tissue sections were then incubated with a goat anti-rabbit horseradish peroxidase (HRP)-conjugated secondary antibody for 1 h at room temperature, followed by colorimetric detection using 3,3’-diaminobenzidine (DAB) (SA1022; Boster, China) per manufacturer’s protocol. Finally, the sections were counterstained with hematoxylin, and ImageJ (version 2.1.0/1.53c^4^) was used to measure and analyze the staining intensity of AOX1 following the published protocol (Crowe and Yue, 2019).

### Clinical Samples

All ccRCC clinical samples were obtained with signed informed consent from the Department of Urology, Renmin Hospital of Wuhan University. This study was approved by the ethical review board of Renmin Hospital of Wuhan University.

### Statistical Analysis

Wherever applicable, a two-tailed Student’s *t*-test, Mann–Whitney *U* test, or Kruskal–Wallis test was used to test the significance of differences between groups. Dunnett’s correction is used to prevent family wise error rates when performing multiple hypothesis tests. Survival analysis was performed with Kaplan–Meier curves using log-rank test. Data were plotted and analyzed using GraphPad Prism 8 (GraphPad Software, CA) and presented as mean ± SEM unless stated elsewhere. We used the receiver operating characteristic (ROC) curve and calculated the area under the curve (AUC) score to show the diagnostic ability of *AOX1* expression in classification of patients. Effect size is expressed as an unstandardized mean difference or the percentage of one group mean to another in this study.

## Results

### Integrative Differential Expression Analysis Across Eight Clear Cell Renal Cell Carcinoma Datasets

**FIGURE 1:**
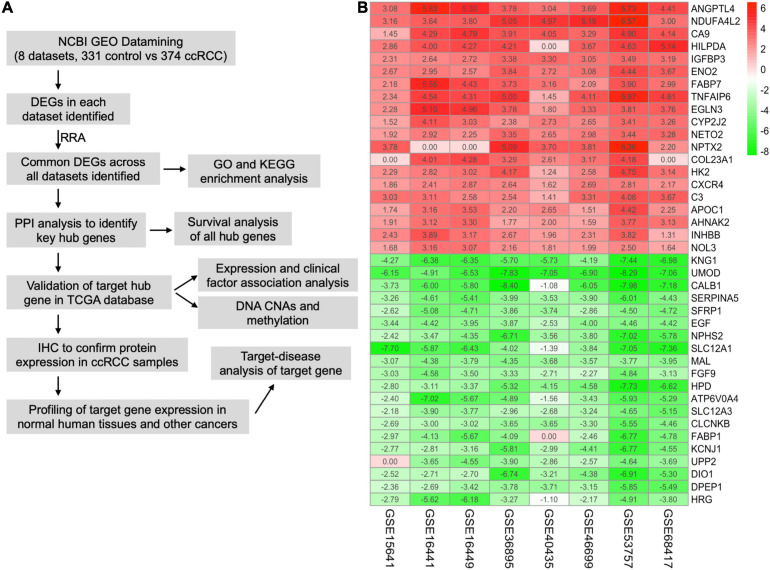
Integrative analysis of multiple clear cell renal cell carcinoma (ccRCC) RNA sequencing datasets. **(A)** Flowchart of this study. **(B)** Heat map showing the top 20 shared upregulated (red) and downregulated (green) differentially expressed genes across all ccRCC datasets. The value in each cell represents log fold change (logFC) of the corresponding gene. RRA, robust rank aggregation.

The workflow of this study is shown in Figure 1A. A comprehensive search in the GEO database yielded eight eligible datasets, namely, GSE15641, GSE68417, GSE46699, GSE53757, GSE40435, GSE36895, GSE16441, and GSE16449. Detailed information about them is shown in Table 1. Eight GEO ccRCC datasets contain 331 normal kidney samples and 374 ccRCC samples in total. Differentially expressed genes in each dataset were first identified using the limma package in R with a cutoff value of absolute log_2_FC > 1 and adjusted *p*-value < 0.05. The differentially expressed genes from each dataset range from 1,000 to 3,500. All differentially expressed genes from the eight datasets were then analyzed using the RobustRankAggreg package (Kolde et al., 2012) to determine the shared differentially expressed genes across all datasets. Among them, 211 and 145 genes were significantly downregulated and upregulated in ccRCC tissues compared with normal kidney tissues. In addition, the top 20 upregulated and downregulated genes can be found in Figure 1B. The volcano plots for the differentially expressed genes are shown in Supplementary Figure 1.

**TABLE 1:** Details of the eligible ccRCC datasets.

References	Accession	Platform	Sample size (Control vs. ccRCC)	Upregulated DEGs	Downregulated DEGs
Jones et al., 2005	GSE15641	GPL96	23:23	517	703
Thibodeau et al., 2009	GSE68417	GPL6244	14:13	725	985
Eckel-Passow et al., 2014	GSE46699	GPL570	63:67	484	522
von Roemeling et al., 2014	GSE53757	GPL570	72:72	1,676	1,585
Wozniak et al., 2013	GSE40435	GPL10558	101:101	489	606
Peña-Llopis et al., 2012	GSE36895	GPL570	23:29	725	870
Liu et al., 2011	GSE16441	GPL6480	17:17	1,485	1,652
Brannon et al., 2010	GSE16449	GPL6408	18:52	1,646	1,776

*ccRCC, clear cell renal cell carcinoma; DEG, differentially expressed gene.*

### Gene Ontology Terms and Kyoto Encyclopedia of Genes and Genomes Pathway Analyses of Differentially Expressed Genes

**FIGURE 2:**
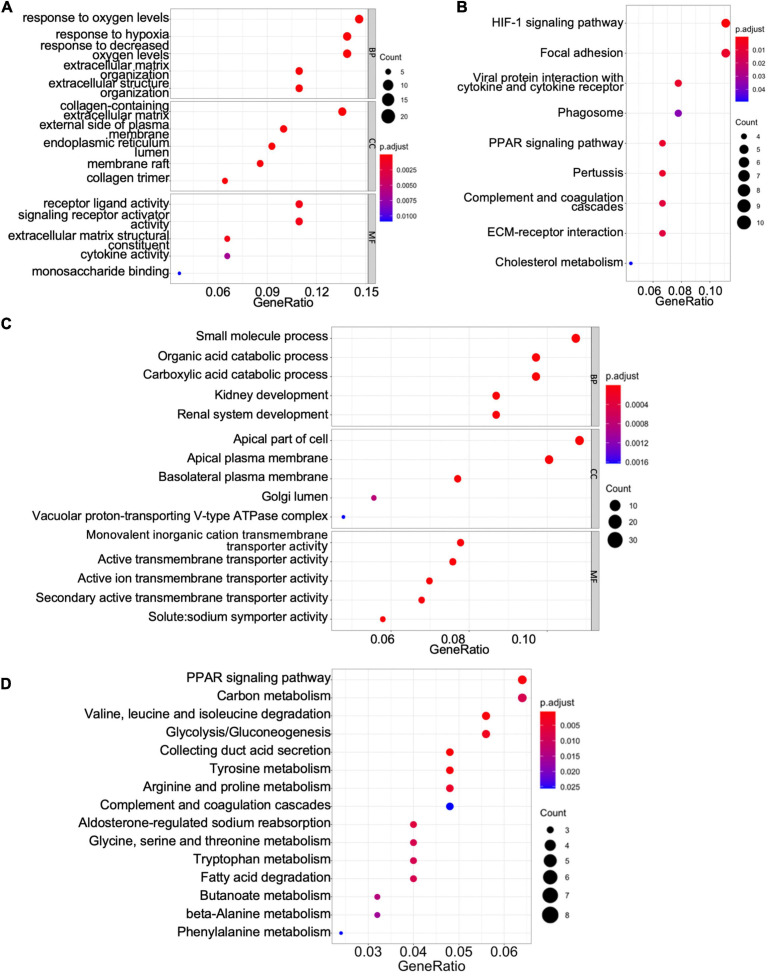
Gene Ontology (GO) term enrichment and Kyoto Encyclopedia of Genes and Genomes (KEGG) pathway analyses of shared differentially expressed genes. **(A,C)** The GO terms of the biological process, cellular component (CC), and molecular function (MF) for upregulated **(A)** and downregulated **(C)** differentially expressed genes. **(B,D)** The KEGG pathway analyses of upregulated **(B)** and downregulated **(D)** differentially expressed genes.

To gain mechanistic and functional insight into the differentially expressed genes between ccRCC tissues and normal kidney tissues, GO term and KEGG enrichment analyses were performed using the clusterProfiler package in R (Yu et al., 2012). The GO hierarchy contains three sub-ontologies: biological process, molecular function, and cellular component. The GO term enrichment analysis reveals that the major biological processes and molecular functions associated with upregulated differentially expressed genes are response to low oxygen levels, extracellular structure organization, receptor–ligand activity, cytokine activity, and monosaccharide binding (Figure 2A). The KEGG pathway analysis shows that the upregulated differentially expressed genes are primarily enriched in the hypoxia-inducible factor (HIF)-1 signaling pathway, focal adhesion, peroxisome proliferator-activated receptor (PPAR) signaling pathway, extracellular matrix–receptor interaction, and cholesterol metabolism (Figure 2B). For downregulated differentially expressed genes, the top biological processes and molecular functions include small-molecule process, organic acid catabolic process, carboxylic acid catabolic process, kidney development, and various transmembrane transporter activities (Figure 2C). Additionally, the downregulated differentially expressed genes are mainly enriched in PPAR signaling pathway, carbon metabolism, collecting duct acid secretion, fatty acid degradation, and diverse amino acid metabolism (Figure 2D). These results suggest that ccRCC cancer cells might better adapt to low oxygen conditions, which confer on them a survival advantage in hypoxic microenvironments in cancer tissue, and altered carbon, organic acid, and various amino acid metabolisms characterize a metabolic reprogramming in ccRCC cells.

### Construction of the Protein–Protein Interaction Network and Identification of Hub Genes

**FIGURE 3:**
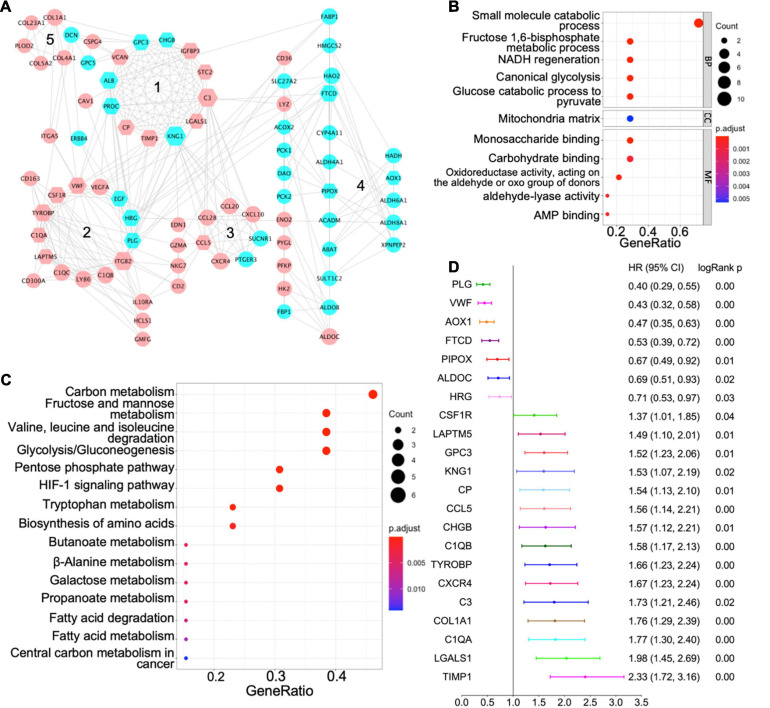
Protein–protein interaction (PPI) network of shared differentially expressed genes and hub genes. **(A)** PPI network of shared differentially expressed genes showing interactions of different functional modules. Blue and pink indicate downregulation and upregulation, respectively. Major hub genes are marked by hexagons. Numbers label different functional modules. **(B)** The Gene Ontology (GO) terms of biological process (BP), cellular component (CC), and molecular function (MF) for functional module 4 in the PPI network. **(C)** The Kyoto Encyclopedia of Genes and Genomes (KEGG) pathway analysis of functional module 4 in the PPI network. **(D)** Forest plot demonstrating survival analyses of major hub genes identified in the PPI network. HR, hazard ratio; CI, confidence interval.

We used the STRING database to establish a PPI network for the shared differentially expressed genes, and the network was processed by Cytoscape software for visualization (Figure 3A). In a PPI network, hub genes are those with more interactions with other nodes, and they are more likely to be essential genes for cells than other genes (Jeong et al., 2001). We identified 42 hub genes in the PPI network of all differentially expressed genes with a cutoff degree of 8, and the top 10 hub genes are *KNG1*, *ITGB2*, *C3*, *TYROBP*, *ALB*, *FTCD*, *VWF*, *PROC*, *HRG*, *TIMP1* (Figure 3A). In addition, five functional modules were identified in the established PPI network by MCODE (Bader and Hogue, 2003), and the genes in each module are listed in Table 2. GO term and KEGG pathway analyses were applied to determine the function of each module (Figures 3B,C for module 4; Supplementary Figure 2 for other modules). The significant biological processes in GO terms and pathways enriched in KEGG analysis for module 4 are related to HIF-1 signaling, glycolysis, amino acid metabolism, and fatty acid metabolism (Figures 3B,C). Module 1 is mainly associated with protein modification and peptide degradation (Supplementary Figure 2A). Module 2 is enriched in cell adhesion, Pap1 pathway, PI3K/Akt pathway, Ras pathway, and mitogen-activated protein kinase (MAPK) pathway (Supplementary Figures 2B,C). Functions of module 3 involve cell chemotaxis, chemokine signaling, tumor necrosis factor (TNF) signaling, interleukin (IL)-17 signaling, Toll-like receptor signaling, and cAMP signaling (Supplementary Figures 2D,E). Module 5 is enriched in extracellular structure modification, glycan catabolism, protein digestion, and PI3K/Akt pathway (Supplementary Figures 2F,G).

**TABLE 2:** Genes in each functional module identified in the PPI network.

Module	Gene
Module 1	*PROC, STC2, TIMP1, VCAN, LGALS1, ALB, KNG1, IGFBP3, CP, GPC3, C3, CHGB*
Module 2	*TYROBP, HRG, C1QA, VEGFA, PLG, CSF1R, ITGB2, EGF, LY86, C1QC, C1QB, VWF, LAPTM5*
Module 3	*SUCNR1, PTGER3, CCL5, CXCL10, CCL28, CXCR4, CCL20*
Module 4	*FBP1, FTCD, HK2, ALDOC, ALDOB, SULT1C2, ALDH6A1, HADH, XPNPEP2, AOX1, ACADM, ABAT, ALDH8A1*
Module 5	*GPC5, DCN, COL5A2, COL1A1, CSPG4, COL4A1, PLOD2, COL23A1*

*PPI, protein–protein interaction.*

Based on the centrality-lethality rule, which implies that the loss of a hub gene is more lethal than the loss of a non-hub protein in a PPI network (He and Zhang, 2006), the hub genes are of great biological significance. We checked the impact of hub genes on survival of ccRCC patients using the Kaplan–Meier plotter (Nagy et al., 2018). Strikingly, 22 out of 42 identified hub genes were associated with better or worse survival in ccRCC patients (Figure 3D), which validates their essential roles as hub genes.

### Validation of AOX1 Dysregulation in Clear Cell Renal Cell Carcinoma

**FIGURE 4:**
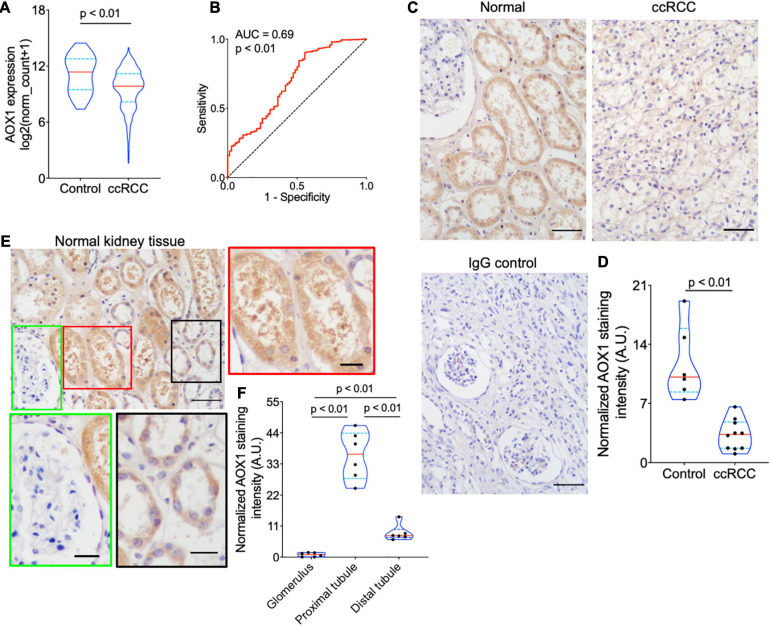
Validation of *AOX1* gene dysregulation in clear cell renal cell carcinoma (ccRCC). **(A)** Violin plot showing significantly lower *AOX1* expression in ccRCC tissues compared with normal control kidney tissues in The Cancer Genome Atlas Kidney Renal Clear Cell Carcinoma (TCGA-KIRC) dataset. *n* = 72 and *n* = 533 for control and ccRCC, respectively. **(B)** Receiver operating characteristic (ROC) curve showing the predictive value of *AOX1* expression for ccRCC patients. AUC, area under the curve. **(C)** Representative images of AOX1 immunohistochemical staining in normal kidney tissues (control) and ccRCC tissues. Scale bar is 50 μm. **(D)** Violin plot for quantification of AOX1 staining intensity in panel **(C)** showing significantly lower AOX1 intensity in ccRCC tissues compared with normal kidney tissues (control). *n* = 6 and *n* = 10 for control and ccRCC, respectively. **(E)** Representative images of AOX1 immunochemical staining in normal kidney tissues demonstrating AOX1 expression in proximal tubules (left panel) and distal tubules (right panel). Scale bar is 50 μm in upper left panel and 20 μm in other panels. **(F)** Violin plot for quantification of AOX1 staining intensity in panel **(E)** shows the strongest AOX1 staining in proximal tubules. *n* = 6 for each group.

*PLG, vWF*, and *AOX1* are the three most protective hub genes for ccRCC patients, and ccRCC patients with a higher expression of these genes have 50% more overall survival advantage over those with a lower expression (Figure 3D). While the roles of PLG and vWF in ccRCC had been well elaborated, AOX1 has not yet been reported. We validated *AOX1* mRNA expression with TCGA-KIRC dataset and AOX1 protein expression with immunohistochemistry. *AOX1* gene expression data from 72 normal kidney tissues and 533 ccRCC tissues in TCGA-KIRC dataset were extracted and analyzed using UCSC Xena (Goldman et al., 2020). The expression of AOX1 mRNA in ccRCC tissues is only 34.2% (95% CI: 23.6–50.0%) as much as that in normal kidney tissues, which confirmed our differential expression analysis (Figure 4A). The ROC curve demonstrates the potential ability of *AOX1* expression level as an indicator to discriminate between normal and ccRCC tissues (AUC = 0.69, *p* < 0.0001) (Figure 4B). In addition, 10 ccRCC clinical samples and six paired normal kidney samples were stained and quantified for AOX1 protein expression. ccRCC patients’ information can be found in Supplementary Table 1. Consistent with the results obtained from differential expression analysis and TCGA-KIRC data mining, AOX1 protein expression in normal kidney tissues is 2.5 times (95% CI: 1.6–3.6) higher than that in ccRCC tissues (Figures 4C,D). We further compared the localization and level of AOX1 protein expression in normal kidney tissues, and the result shows that proximal tubules have the most AOX1 protein expression, followed by distal tubules, while the glomeruli have the least AOX1 protein expression (Figures 4E,F).

### High AOX1 mRNA Expression Denotes Better Survival in Clear Cell Renal Cell Carcinoma

**FIGURE 5:**
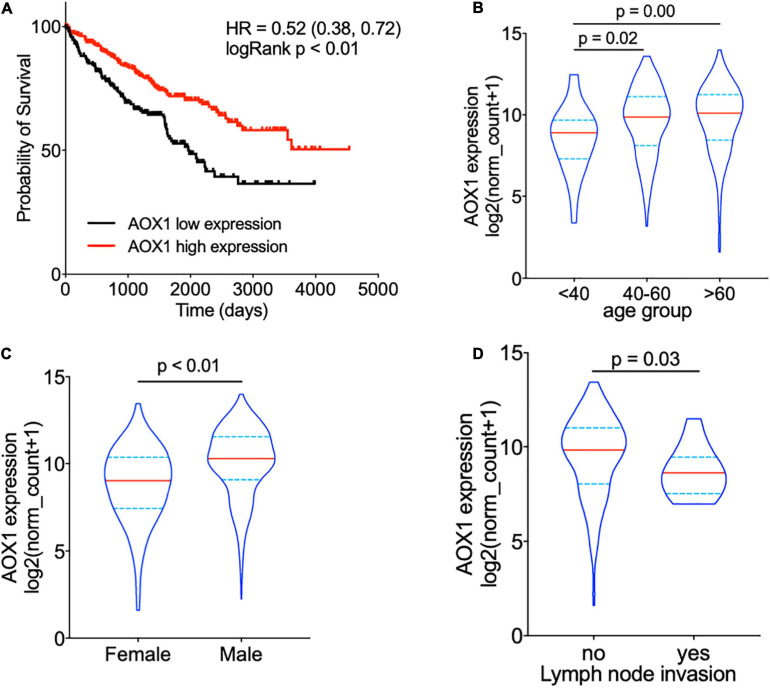
*AOX1* expression level correlates with overall survival and clinical factors of clear cell renal cell carcinoma (ccRCC) patients. **(A)** Kaplan–Meier curve showing ccRCC patients with higher *AOX1* expression have better overall survival. *AOX1* expression level was defined as low or high expression based on the mean of expression. HR, hazard ratio. **(B)** Violin plot showing significantly lower *AOX1* expression in younger (<40 years old) ccRCC patients compared with older (40–60 or >60 years old) ccRCC patients. *n* = 26, *n* = 235, and *n* = 265 for <40, 40–60, and >60 groups, respectively. **(C)** Quantification of *AOX1* expression in different gender groups of ccRCC patients showing significantly lower *AOX1* expression in female ccRCC patients. *n* = 185 and *n* = 341 for female and male, respectively. **(D)** Violin plot of *AOX1* expression in ccRCC patients with (yes) or without (no) lymph node invasion. *n* = 236 and *n* = 13 for with or without lymph node invasion, respectively.

To understand the effect of AOX1 on the prognosis of ccRCC patients, 516 eligible ccRCC patients from TCGA-KIRC dataset were subgrouped into two groups based on *AOX1* mRNA expression in UCSC Xena (Goldman et al., 2020). Kaplan–Meier curve analysis was performed to compare the overall survival between the two groups. The result demonstrates that ccRCC patients with low *AOX1* mRNA expression levels had worse overall survival than those with high *AOX1* mRNA expression levels [high expression vs. low expression hazard ratio (HR): 0.52, 95% CI: 0.38–0.72] (Figure 5A). To clarify the definite relationship of *AOX1* with different clinical factors of ccRCC, we estimated the mRNA expression levels of *AOX1* in different patient subgroups. ccRCC patients aged between 40 and 60 years and older than 60 years have 2.1 times (95% CI: 1.2–3.9) and 2.3 times (95% CI: 1.3–4.2) higher *AOX1* mRNA expression than that in patients younger than 40 years, respectively (Figure 5B). In addition, male ccRCC patients express 2.4 times more *AOX1* mRNA (95% CI: 1.9–3.1) than female patients (Figure 5C). Higher histologic grade and lymph node invasion often correlate with poor prognosis and relapse. Moreover, ccRCC patients with lymph node invasion also have lower *AOX1* expression levels (Figure 5D). No difference was found in *AOX1* expression levels in ccRCC patients with respect to pathologic stages, metastasis, smoking, or histologic grade (Supplementary Figure 3). To investigate the effect of different factors on ccRCC patients’ overall survival, we performed Cox regression analyses. Cox regression confirmed that higher AOX1 mRNA expression (HR: 0.89, 95% CI: 0.83–0.96) has a protective role. In addition, higher pathologic stage (HR: 1.68, 95% CI: 1.43–1.96), higher histologic grade (HR: 1.53, 95% CI: 1.20–1.94), and older patients (HR: 1.03, 95% CI: 1.02–1.05) are associated with worse overall survival (Table 3).

**TABLE 3:** Cox regression analyses.

Variable	Unadjusted model			Adjusted model		
	HR	95% CI	*p*-value	HR	95% CI	*p*-value
*AOX1*	0.90	(0.84–0.95)	<0.01	0.89	(0.83–0.96)	0.00
Male	0.94	(0.68–1.28)	0.68	1.12	(0.79–1.59)	0.53
Pathologic stage	1.88	(1.64–2.15)	<0.01	1.68	(1.44–1.96)	<0.01
Histologic grade	2.33	(1.89–2.87)	<0.01	1.53	(1.20–1.94)	<0.01
Age	1.03	(1.01–1.04)	<0.01	1.03	(1.02–1.05)	<0.01

*HR, hazard ratio.*

### Potential Genetic Alterations Associated With AOX1 Dysregulation

**FIGURE 6:**
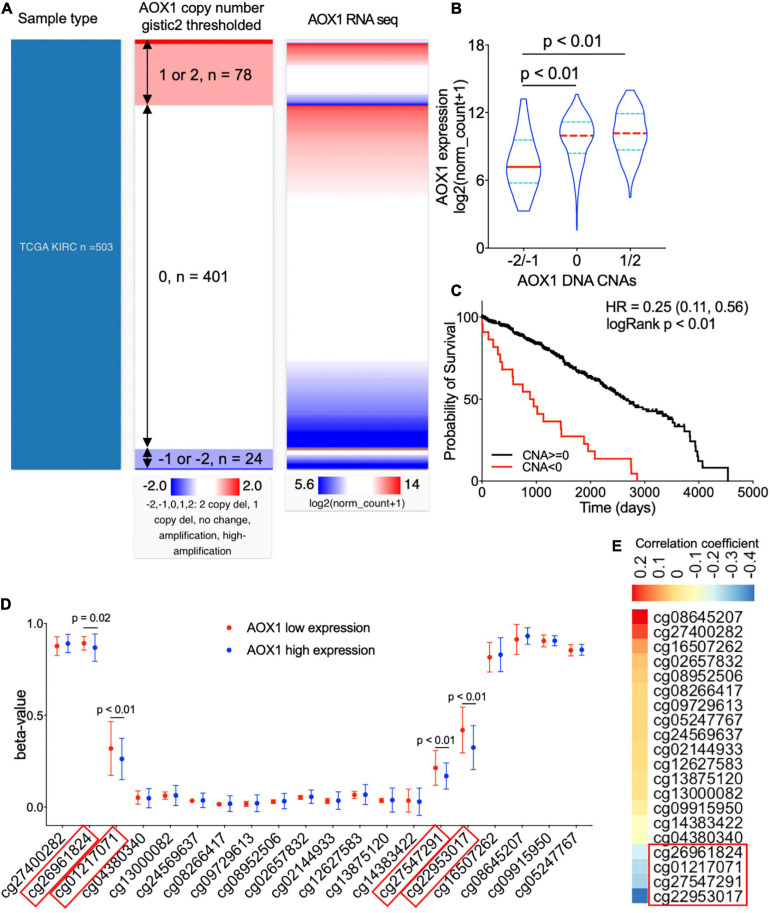
Genetic alterations of *AOX1* gene in clear cell renal cell carcinoma (ccRCC) patients. **(A)** Heat map showing the correlation between *AOX1* DNA copy number alteration (CNA) and its expression. **(B)** Violin plot showing *AOX1* expression in different CNA groups. CNAs were defined as follows: no change (0), heterozygous deletion (–1), homozygous deletion (–2), amplification (1), and high amplification (2). *n* = 23, *n* = 401, and *n* = 78 for –2/–1, 0, and 1/2 groups respectively. **(C)** Kaplan–Meier curve of ccRCC patients subgrouped by *AOX1* DNA CNAs shows worse overall survival in ccRCC patients with copy number deletions in the *AOX1* gene. *n* = 23 and *n* = 479 for CNA < 0 and CNA ≥ 0, respectively. **(D)** Methylation levels (beta-value) of CpG site in *AOX1* DNA locus subgrouped by *AOX1* expression level. *n* = 125 and *n* = 177 for *AOX1* low expression and high expression, respectively. **(E)** Heat map showing the correlation between *AOX1* expression and CpG site methylation level of *AOX1* DNA. Color bar indicates Pearson’s correlation coefficient.

To understand the potential mechanism of *AOX1* gene dysregulation in ccRCC, we examined the DNA CNAs of the *AOX1* gene in ccRCC. DNA CNAs are somatic variations that lead to duplication or deletion of certain sections of the chromosome and are quite common in human cancers (Tang et al., 2017). In TCGA-KIRC database, DNA CNA data were available for 503 ccRCC patients. Among them, 24 have either *AOX1* homozygous deletion (–2) or heterozygous deletion (–1), and 78 of them have either *AOX1* low-level (1) or high-level (2) amplification (Figure 6A). We checked the correlation between *AOX1* mRNA expression and its DNA CNAs. Although *AOX1* DNA amplification (1/2) has no impact on its mRNA expression, *AOX1* DNA deletion (–1/–2) is associated with significantly lower *AOX1* mRNA expression (Figure 6B), with only 22.2% (95% CI: 12.1–80.8%) *AOX1* mRNA expression as much as that of *AOX1* DNA normal (0) group. Kaplan–Meier survival curve shows that the ccRCC patient subgroup without *AOX1* DNA deletion (CNA ≥ 0) has a survival advantage over that with *AOX1* DNA deletion (CNA < 0) (CNA ≥ 0 vs. CNA < 0 HR: 0.25, 95% CI: 0.11–0.56) (Figure 6C). DNA methylation is another epigenetic tool to regulate gene expression in eukaryotic cells; therefore, we compared the methylation status of 20 CpG sites of the *AOX1* gene in ccRCC patients subgrouped by *AOX1* expression. Four out of the 20 examined CpG sites have significantly higher methylation levels in ccRCC patients with lower *AOX1* mRNA expression (Figure 6D), and the methylation levels of these four CpG sites are negatively correlated with *AOX1* mRNA expression (Figure 6E). All in all, these results imply that hypermethylation and copy number deletion may cause the downregulation of *AOX1* in ccRCC.

### Expression Profile of AOX1 in Normal and Cancer Tissues

**FIGURE 7:**
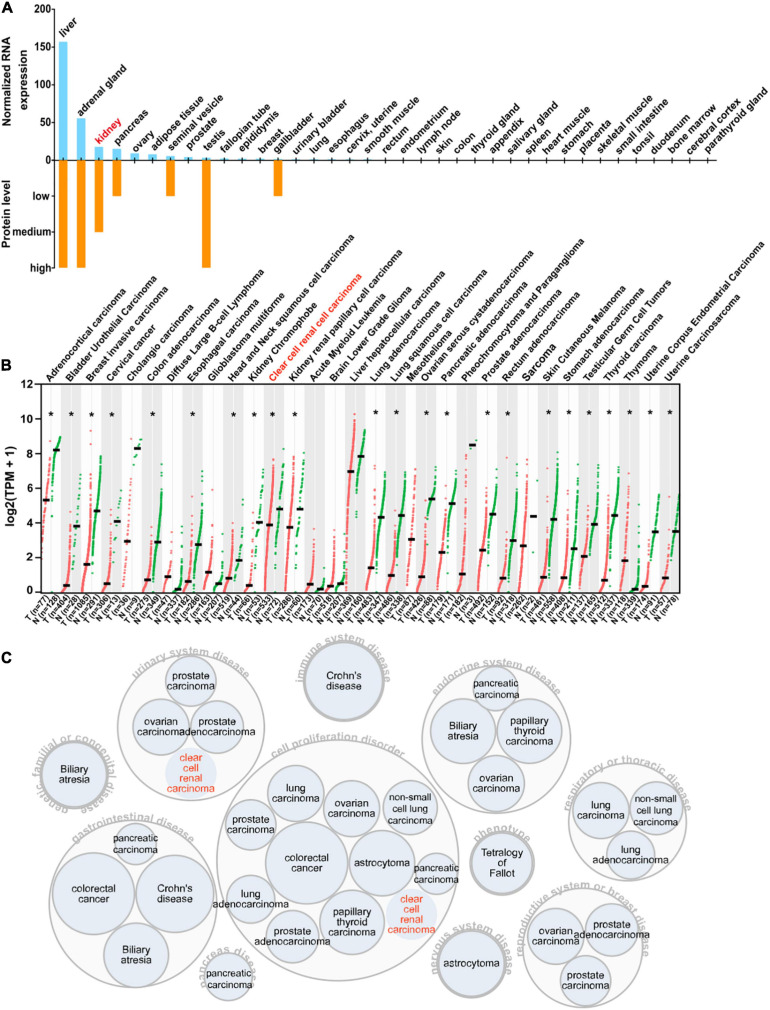
Expression profile of AOX1. **(A)** Bar graph depicting *AOX1* mRNA and protein expression in 37 human tissues in the Human Protein Atlas (HPA) database. **(B)** Comparison of *AOX1* expression in 31 different kinds of cancers and matched normal control samples. T stands for tumor, and N stands for normal. Clear cell renal cell carcinoma (ccRCC) patient data were extracted from The Cancer Genome Atlas Kidney Renal Clear Cell Carcinoma (TCGA-KIRC) using UCSC Xena. *p < 0.05. **(C)** Target–disease association analysis revealing AOX1-associated diseases.

To acquire a general knowledge on AOX1 expression in normal tissue, we queried the HPA database for the *AOX1* gene. The HPA database offers thorough mRNA and protein expression profiles of AOX1 in 37 normal tissue types, and most of the examined tissues have low to no *AOX1* mRNA or protein expression (Figure 7A). Among all the listed tissue types, liver, adrenal gland, testis, and kidney have the most abundant AOX1 protein expression, while liver, adrenal gland, kidney, and pancreas are the four tissues with the highest *AOX1* mRNA expression level. Next, we compared *AOX1* mRNA expression in 31 different types of tumor samples and normal tissue samples using GEPIA (Tang et al., 2017). *AOX1* mRNA expression is significantly dysregulated in 23 out of 31 types of tumors, and one of the 23 types of tumors has upregulated *AOX1* level, while 22 of the 23 types of tumors have downregulated *AOX1* levels, including ccRCC (Figure 7B). We explored the potential diseases associated with *AOX1* gene on the Open Target Platform (Carvalho-Silva et al., 2019) and identified 11 different kinds of diseases related to *AOX1* gene dysregulation (Figure 7C). The disease spectrum covers almost all the major systems in the human body, including urinary system diseases, genetic familial or congenital diseases, immune system diseases, endocrine system diseases, respiratory or thoracic diseases, gastrointestinal diseases, pancreas diseases, cell proliferation disorders, nervous system diseases, and reproductive system diseases. The identified diseases are mostly cancers, and ccRCC is among them (Figure 7C). To conclude, AOX1 is abundant in normal kidney tissue, and it is downregulated not only in ccRCC but also in many other cancers.

## Discussion

ccRCC is the most prevalent kidney cancer and caused 179,368 deaths worldwide in 2020. In this study, by integrative analysis of eight different ccRCC RNA-seq datasets, we identified 356 common differentially expressed genes between ccRCC tissues and normal kidney tissues across eight ccRCC sequencing datasets. We demonstrated that the differentially expressed genes are mainly enriched in adaptation to hypoxic condition and metabolic reprogramming. Among the 356 differentially expressed genes, AOX1 is identified as a prognostic factor for ccRCC patients. ccRCC tissues have significantly lower *AOX1* mRNA and protein expression than normal kidney tissues, and lower expression in tumor tissues was associated with worse overall survival in ccRCC patients. *AOX1* expression is associated with several major ccRCC clinical factors, including age, gender, and lymph node invasion. Then, we proposed that *AOX1* gene dysregulation in ccRCC patients might result from *AOX1* DNA deletion and hypermethylation. In addition, we profiled *AOX1* expression patterns in RNA and protein levels in distinct normal and cancerous tissues and found consistent downregulation of the *AOX1* gene in many other cancer types.

Although massive ccRCC RNA-seq data have been produced over the past 20 years, the presence of heterogeneity between different studies makes it difficult to integrate all the datasets and screen for the common differentially expressed genes. The robust rank aggregation method (Kolde et al., 2012) provides a solution to this problem. Since it was first published in 2012, the robust rank aggregation method has been cited 298 times as of September/October 2020, which is enough to place it in the top 1% of the academic field of biology and biochemistry. Moreover, differentially expressed genes identified in our integrative analysis using robust rank aggregation overlap with differentially expressed genes in other studies (Xu et al., 2019; Zhang N. et al., 2020; Zhang F. et al., 2020), which confirms the effectiveness and accuracy of our analysis.

Enrichment analysis shows that the differentially expressed genes’ principal dysregulated pathways and biological processes involve response to low oxygen levels, HIF-1 signaling pathway, cholesterol metabolism, carbon metabolism, fatty acid degradation, amino acid metabolism, organic acid catabolic process, and carboxylic acid catabolic process. These results suggest an increased adaptation to hypoxia and the metabolic reprogramming of cancer cells to tumor microenvironment, known as the Warburg effect (Liberti and Locasale, 2016). At least 90% of ccRCC tumors are characterized by loss of von Hippel-Lindau (VHL) tumor suppressor function (Ricketts and Linehan, 2018), and VHL mediates proteasomal degradation of the HIF α-subunit (Schödel et al., 2016). While most solid tumors feature hypoxia or shortage of oxygen and blood supply in the tumor microenvironment as a result of the abnormal, rapid, and uncontrolled proliferation of tumor cells (Jing et al., 2019), the Warburg effect confers better flexibility on ccRCC cells to hypoxia and lack of nutrition. The fact that AOX1-related functional modules in the PPI are mainly related to HIF-1 signaling, glycolysis, amino acid metabolism, and fatty acid metabolism further implies a role of AOX1 in metabolic reprogramming. On the other hand, hypoxia induces posttranslational modification to reprogram metabolism in ccRCC to better support the survival of tumor cells (Bacigalupa and Rathmell, 2020). Loss of AOX1 in advanced bladder cancer also leads to alteration in metabolism (Vantaku et al., 2020), consistent with our findings in ccRCC.

AOX1 protein expression is most abundant in the liver, adrenal gland, and testis, with medium expression in the kidney. In a normal kidney, it is mainly localized to the proximal tubular epithelium, from which the ccRCC originates. The proximal tubule reabsorbs approximately 65% of the NaCl filtered through the glomerulus, heavily relying on the Na+/H+ exchangers on the lumen side of the proximal tubule. Meanwhile, Na+/H+ exchanger 3 activity can be modified by superoxide, and fluid reabsorption is fine-tuned by redox balance in proximal tubule epithelium (Panico et al., 2009). Preferential expression of AOX1 in the proximal tubule contributes to the production of various reactive oxygen species, which might regulate reabsorption in the proximal tubule.

Decreased *AOX1* mRNA and protein levels in ccRCC tissues compared with normal tissues suggest a tumor-suppressive role of AOX1. And this effect is not restricted to ccRCC but universally exists in many other types of tumors, considering the significantly diminished expression of *AOX1* in more than half of the tumor types in TCGA database. In the ccRCC patients, reduced expression of *AOX1* predicts worse overall survival, and the predictive value is further supported by the target–disease association analysis of AOX1, which identified more than 20 kinds of cancers as its potentially relevant diseases including ccRCC. We analyzed *AOX1* expression in ccRCC patients subgrouped by factors that may affect ccRCC patients’ prognosis, including age, gender, histologic grade, lymph node invasion, metastasis, pathologic stage, and smoking status. Our results showed that younger (<40 years old) and female patients with lymph node invasion have significantly lower *AOX1* expression. Besides, our Cox regression analyses revealed that ccRCC patients with higher *AOX1* expression have better overall survival, which further confirmed the survival analysis. In addition, younger age, lower histologic stage, and lower pathologic grade are associated with better survival, which is consistent with the general knowledge on ccRCC. CNAs are common genetic modifications contributing to gene dysregulation in ccRCC (Thiesen et al., 2017). Examples of the previously reported tumor suppressor gene CNAs in ccRCC are 2p21.31 (RBM6, RBM5, LIMD1, and TCTA) and 3p22.2 (MLH1) (Thiesen et al., 2017). More than half of invasive cervical cancers are characterized by deletions of 2q33-q37 (Rao et al., 2004), and the *AOX1* gene is located in 2q33.1 in the human genome. This intrigued us to examine the relationship between *AOX1* CNA status and its expression in ccRCC. We found that *AOX1* DNA deletion is associated with lower *AOX1* expression in ccRCC, suggesting that DNA CNA might be a potential mechanism of AOX1 downregulation in ccRCC.

On the other hand, *AOX1* DNA amplification has no effect on its expression, which is a shred of evidence that not all CNAs can translate into gene expression alterations (Bhattacharya et al., 2020). A previous study reported downregulation of *AOX1* in advanced bladder cancer due to methylation modification by EZH2 (Vantaku et al., 2020). Similarly, we observed significantly higher methylation levels in four CpG sites (cg26961824, cg01217071, cg27547291, and cg22953017) of the *AOX1* gene in ccRCC with lower *AOX1* expression levels compared with ccRCC with higher *AOX1* expression levels, and the methylation level shows a moderate negative correlation with the *AOX1* gene expression. Accordingly, we conclude that methylation modification might be another important mechanism for suppressed AOX1 expression in ccRCC.

The study may have some limitations. Although we integrated several ccRCC datasets, the sample size is still small, large and carefully designed preclinical studies are needed to confirm AOX1’s effect on ccRCC before clinical implementation. Heterogeneity between different studies is unavoidable, although the robust rank aggregation method was implemented to minimize their effects. Further validation of differential expression by qPCR might be necessary. In addition, we have not clarified the mechanism through which AOX1 suppresses ccRCC progression in this study. Our future research may focus on the molecular mechanism for AOX1’s dysregulation in ccRCC and how its expression impacts the behavior and biology of cancer cells by using transgenic mouse and cell lines.

## Conclusion

To conclude, by integrative analysis of multiple ccRCC RNA-seq datasets, we identified *AOX1* as a suppressor gene for ccRCC, which is downregulated in ccRCC tissues compared with normal kidney tissues, and low *AOX1* expression predicts worse overall survival in ccRCC patients. Downregulation of *AOX1* gene in ccRCC might result from *AOX1* DNA deletion and hypermethylation.

## Data Availability Statement

The original contributions presented in the study are included in the article/Supplementary Material, further inquiries can be directed to the corresponding author/s.

## Ethics Statement

The studies involving human participants were reviewed and approved by the ethical review board of Renmin Hospital of Wuhan University. The patients/participants provided their written informed consent to participate in this study.

## Author Contributions

LX conceived the project, analyzed the data, and wrote the manuscript. YF and JT performed the experiments. WH collected and processed the samples. WH, HW, and SL conceived the project, revised the manuscript, and secured the funding. All authors reviewed and approved the final version of the manuscript.

## Conflict of Interest

The authors declare that the research was conducted in the absence of any commercial or financial relationships that could be construed as a potential conflict of interest.

## Footnotes

1 https://string-db.org/

2 https://cytoscape.org/

3 http://www.proteinatlas.org

4 https://imagej.nih.gov/ij/

## Supplementary Material

Supplementary Figure 1 Volcano plots showing differentially expressed genes in each dataset. Red and green dots represent upregulated and downregulated genes respectively.

Supplementary Figure 2 GO term enrichment and KEGG pathway analyses of differentially expressed genes in different functional modules in PPI network. GO terms of module 1 **(A)**, module 2 **(B)**, module 3 **(D)**, and module 5 **(G)**. BP, biological process. CC, cellular component. MF, molecular function. KEGG pathway analyses of module 2 **(C)**, module 3 **(E)**, and module 5 **(F)**.

Supplementary Figure 3 *AOX1* expression in ccRCC patient subgroups. No differences were observed between ccRCC patients subgrouped by pathologic stages **(A)**, metastatic states **(B)**, smoking history **(C)**, and histological grades **(D)**. *n* = 265, 55, 122, and 84 for stage I, II, III, and IV in **(A)**. *n* = 417 and 78 for M0 and M1 in **(B)**. *n* = 46 and 39 for non-smoker and smoker group in **(C)**, *n* = 14, 228, 206, 75, 242, and 281 for G1, G2, G3, G4, G1/G2, and G3/G4 in **(D)**.

Supplementary Table 1 Detailed information of ccRCC patients.
