# Supplementary figures and images for: Expression of AOX1 Predicts Prognosis of Clear Cell Renal Cell Carcinoma
Source: Front Genet. 2021 Jul 5;12:683173. doi: 10.3389/fgene.2021.683173 (PMC8287305; doi:10.3389/fgene.2021.683173)

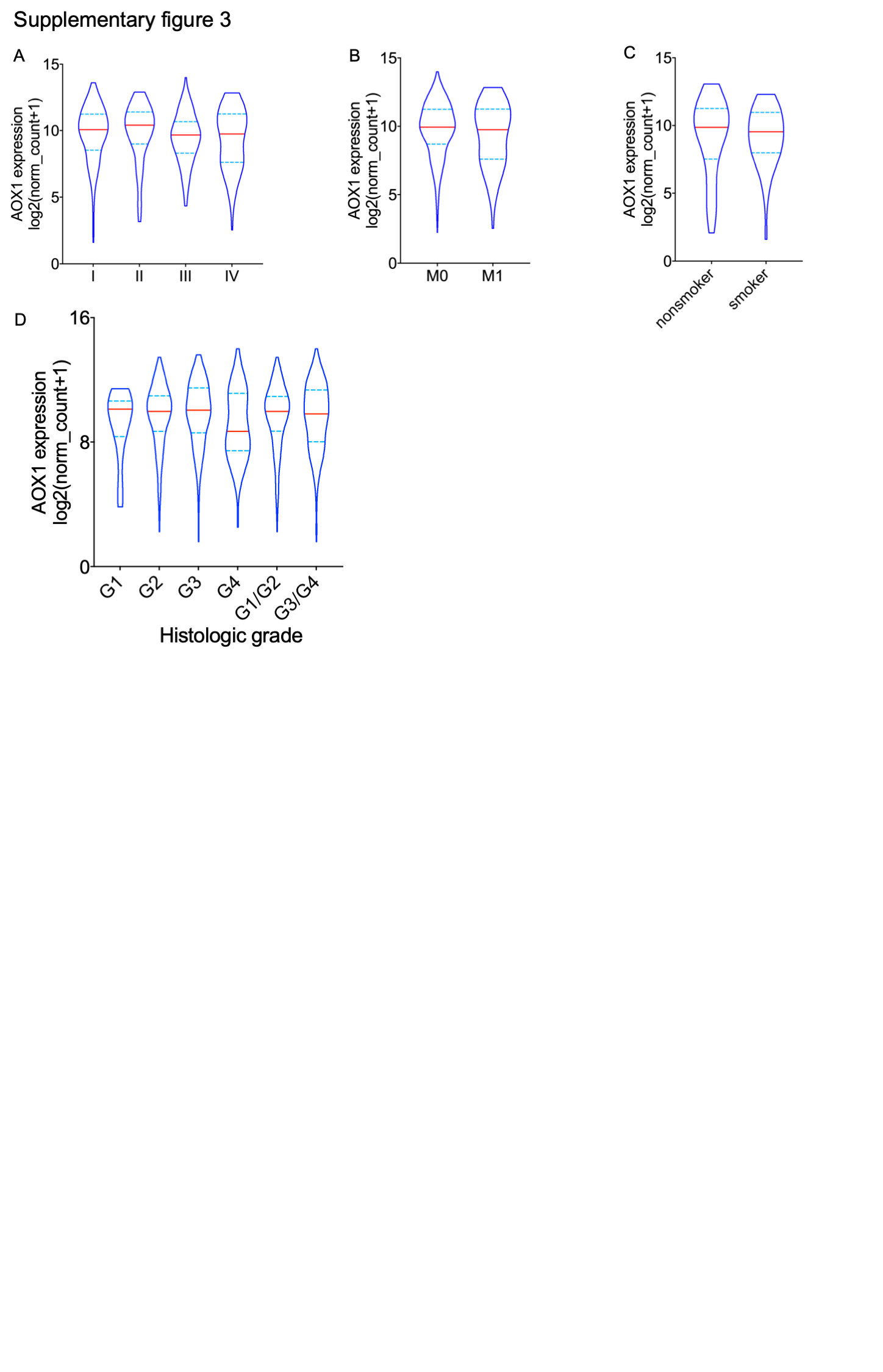

Supplement: Supplementary Figure 1 — Volcano plots showing differentially expressed genes in each dataset. Red and green dots represent upregulated and downregulated genes respectively. [file Data_Sheet_1.zip › supplementary files/Supplementary figure 3.tiff]

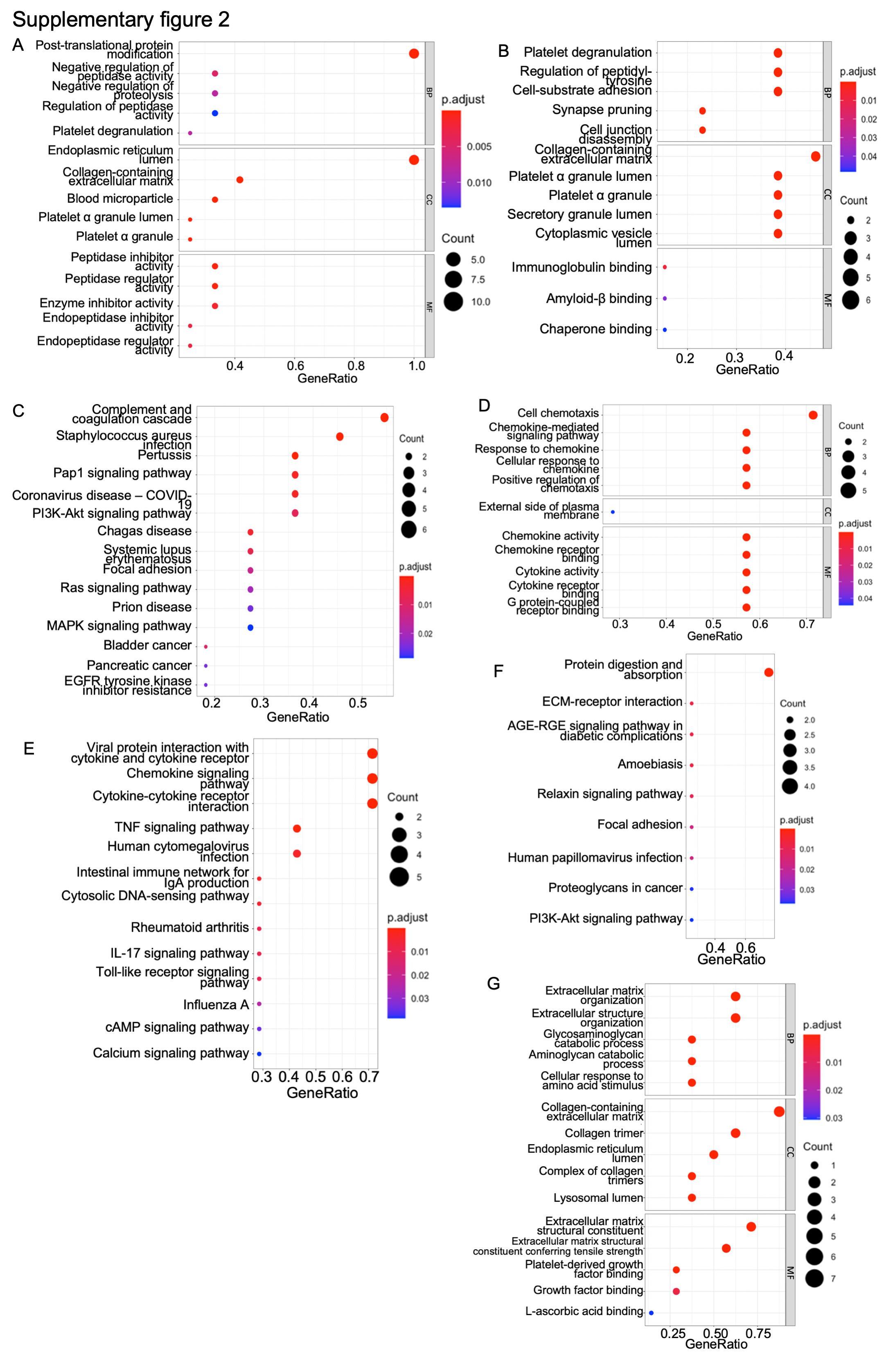

Supplement: Supplementary Figure 1 — Volcano plots showing differentially expressed genes in each dataset. Red and green dots represent upregulated and downregulated genes respectively. [file Data_Sheet_1.zip › supplementary files/Supplementary figure 2.tiff]

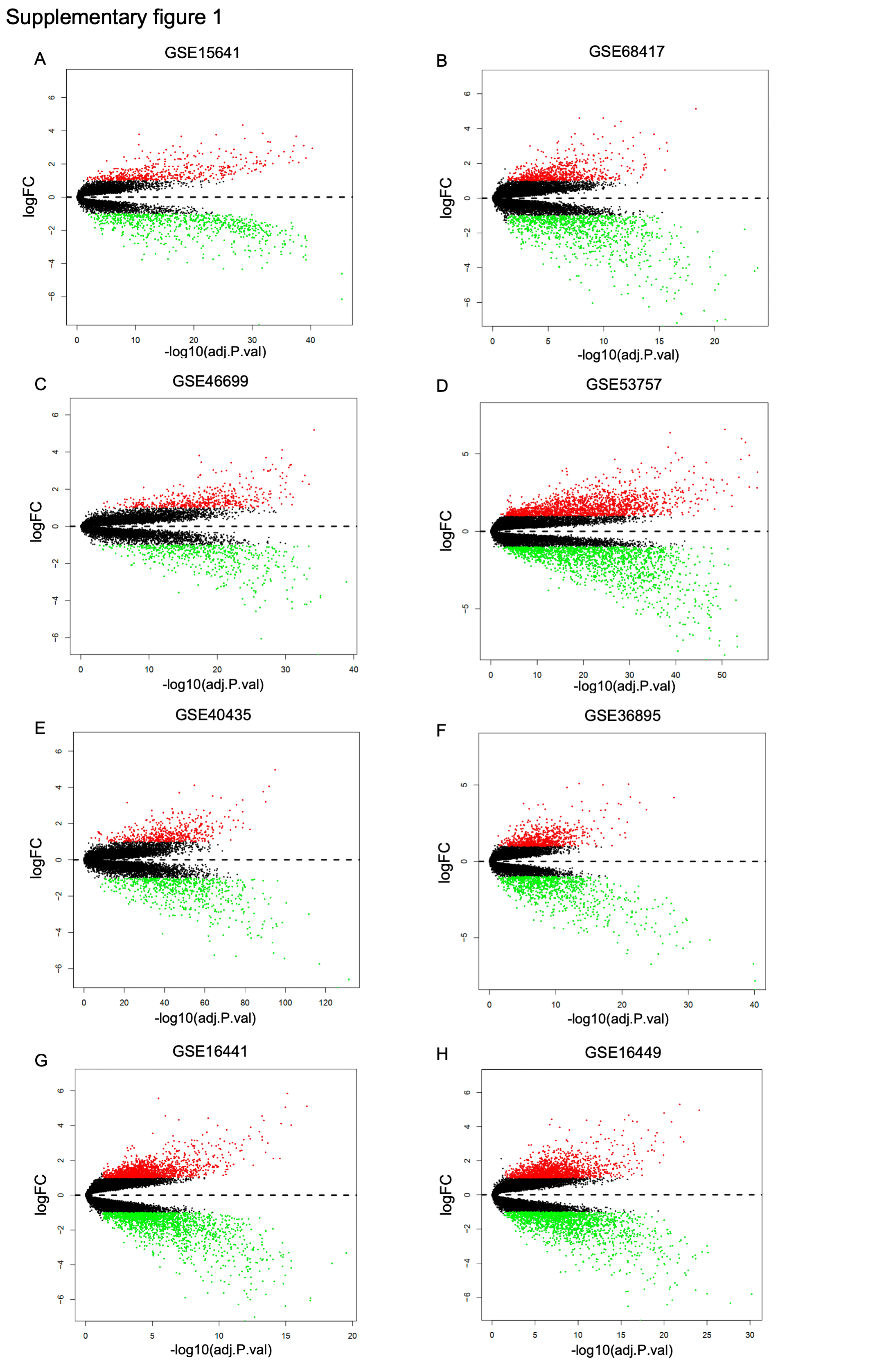

Supplement: Supplementary Figure 1 — Volcano plots showing differentially expressed genes in each dataset. Red and green dots represent upregulated and downregulated genes respectively. [file Data_Sheet_1.zip › supplementary files/Supplementary figure 1.tiff]

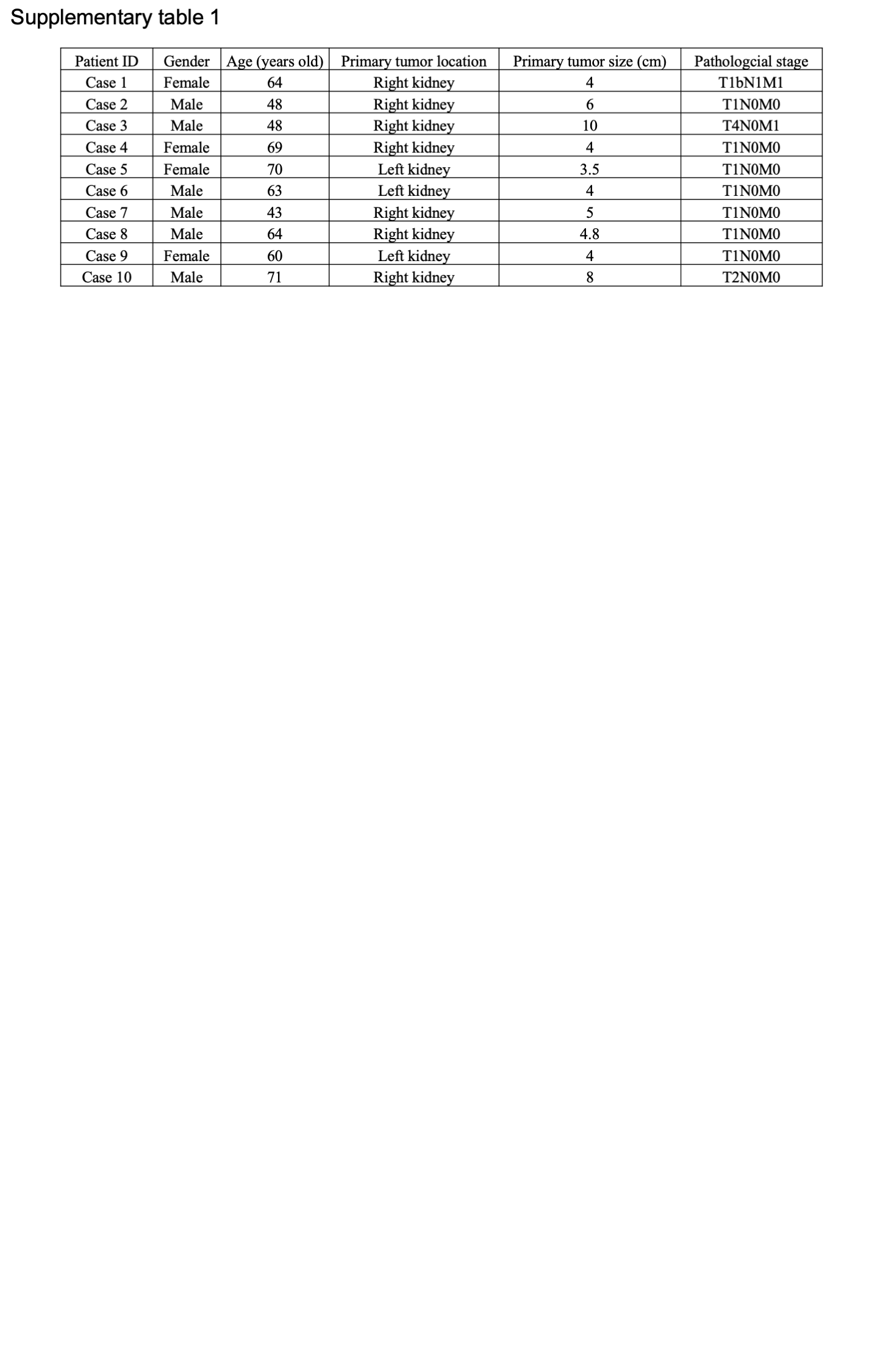

Supplement: Supplementary Figure 1 — Volcano plots showing differentially expressed genes in each dataset. Red and green dots represent upregulated and downregulated genes respectively. [file Data_Sheet_1.zip › supplementary files/Supplementary table 1.tiff]
